# Epilepsia en Pacientes con Angiopatía de Moyamoya

**DOI:** 10.31083/RN37504

**Published:** 2025-10-30

**Authors:** Mario Bautista-Lacambra, Vanesa Garayoa-Irigoyen, Luisa-Fernanda Tique-Rojas, María Seral-Moral, Jesús Moles-Herbera, Amparo López-Lafuente, Rosario Barrena-Caballo, Carlos Tejero-Juste, Marta Palacín-Larroy, Herbert Tejada-Meza

**Affiliations:** ^1^Servicio de Neurología, Hospital Universitario Miguel Servet, 50009 Zaragoza, España; ^2^Grupo de Investigación en Neurociencias, Instituto de Investigación Sanitaria (IIS) Aragón, 50009 Zaragoza, España; ^3^Departamento de Medicina, Dermatología y Psiquiatría de la Universidad de Zaragoza, 50009 Zaragoza, España; ^4^Unidad de Epilepsia Fármaco-Resistente, Hospital Universitario Miguel Servet, 50009 Zaragoza, España; ^5^Servicio de Neurología, Hospital Universitario San Jorge, 22004 Huesca, España; ^6^Servicio de Neurocirugía, Hospital Universitario Miguel Servet, 50009 Zaragoza, España; ^7^Sección de Neuropediatría y Neurometabolismo, Servicio de Pediatría del Hospital Universitario Miguel Servet, 50009 Zaragoza, España; ^8^Unidad de Neurorradiología Intervencionista del Servicio de Radiología del Hospital Universitario Miguel Servet, 50009 Zaragoza, España; ^9^Servicio De Neurología, Hospital Clínico Universitario Lozano Blesa, 50009 Zaragoza, España; ^10^Sección de Neurovascular, Servicio de Neurología, Hospital Universitario Miguel Servet, 50009 Zaragoza, España

**Keywords:** enfermedad de moyamoya, síndrome de moyamoya, angiopatía de moyamoya, epilesia, crisis, epilepsia fármaco-resistente, tratamiento, moyamoya disease, moyamoya syndrome, moyamoya angiopathy, epilepsy, seizures, drugresistant epilepsy, treatment

## Abstract

**Introducción:**

La angiopatía de moyamoya es una enfermedad cerebrovascular caracterizada por la estenosis progresiva de las carótidas internas intracraneales. Existe poca bibliografía que aborde la epilepsia en esta entidad, especialmente en países occidentales.

**Metodología:**

Estudio retrospectivo de los hospitales públicos de Aragón donde se analizan los datos de todos los pacientes diagnosticados con angiopatía de moyamoya entre 1981–2024. Se estudiaron aspectos epidemiológicos de la enfermedad, así como la presencia de epilepsia y su manejo en este grupo de pacientes.

**Resultados:**

Se incluyeron 26 pacientes, con una prevalencia estimada en Aragón de 1,71 casos por 100.000 habitantes. La edad media al diagnóstico fue de 36,64 años, con una distribución equitativa por sexo. La mitad de los pacientes presentaban un síndrome de moyamoya. El 50% de los pacientes padeció una crisis epiléptica y el 42,31% del total cumplió criterios diagnósticos de epilepsia. La mayor parte de las crisis fueron focales (81,8%), con predominio de semiología de lóbulo frontal. El levetiracetam fue el tratamiento más utilizado. Hasta cuatro de los doce pacientes con epilepsia cumplían criterios diagnósticos de epilepsia fármaco-resistente.

**Conclusiones:**

Si bien la prevalencia de moyamoya en nuestra serie es menor a las asiáticas, la prevalencia de la epilepsia fue mucho mayor (50% de pacientes con crisis vs 0,9–18,9% de series asiáticas). En nuestra serie, existe relación entre la epilepsia en la angiopatía de moyamoya y la aparición de síncopes, deterioro cognitivo, trastorno afectivo y un diagnóstico a edad más temprana. No se dispone de otros estudios que aborden porcentaje de epilepsias fármaco-refractarias en estos pacientes.

## Abstract (en)

**Introduction:**

Moyamoya angiopathy is a cerebrovascular disease characterized by progressive stenosis of the intracranial internal carotid arteries. There is limited literature addressing epilepsy in this condition, especially in Western countries.

**Methodology:**

This was a retrospective study conducted in the public hospitals of Aragón, analyzing data from all patients diagnosed with moyamoya angiopathy between 1981 and 2024. Epidemiological aspects of the disease were studied, as well as the presence of epilepsy and its management in this group of patients.

**Results:**

A total of 26 patients were included, with an estimated prevalence in Aragón of 1.71 cases per 100,000 inhabitants. The mean age at diagnosis was 36.64 years, with an equal sex distribution. Half of the patients presented with moyamoya syndrome. Fifty percent of the patients experienced a seizure and 42.31% of the total met diagnostic criteria for epilepsy. Most seizures were focal (81.8%), with a predominance of frontal lobe semiology. Levetiracetam was the most commonly used treatment. Up to four of the twelve patients with epilepsy met diagnostic criteria for drug-resistant epilepsy.

**Conclusions:**

Although the prevalence of moyamoya in our series was lower than in Asian populations, the prevalence of epilepsy was significantly higher (50% of patients with seizures vs 0.9–18.9% in Asian series). In our cohort, epilepsy in moyamoya angiopathy was associated with the occurrence of syncope, cognitive impairment, affective disorders, and an earlier age at diagnosis. There are no other studies available addressing the percentage of drug-resistant epilepsy in these patients.

## 1. Introducción

La angiopatía de moyamoya (MMA) se produce por una estenosis progresiva de la porción intracraneal de las carótidas internas. Dentro de la MMA nos encontramos la enfermedad de moyamoya (MMD) cuando no existe una causa para este proceso y síndrome de moyamoya (MMS) cuando existe una causa claramente descrita para favorecer la enfermedad [1].

Se trata de una entidad más frecuente en países orientales, con una prevalencia de 10,5 casos por 100.000 habitantes en Japón y 16,1 casos por 100.000 habitantes en Corea del Sur [2]. La prevalencia en países occidentales ha sido poco estudiada y se estima en 0,3 casos por 100.000 habitantes en Europa y de 0,086 casos por 100.000 personas entre adultos norteamericanos [3, 4].

La MMA se acompaña de múltiples síntomas consecuencia de esa estenosis progresiva, como ictus isquémico, ictus hemorrágico, trastornos cognitivo-conductuales o epilepsia, un aspecto poco abordado en las guías sobre esta entidad [4].

Para el diagnóstico de la MMA, según las recomendaciones de las Guías Japonesas de 2021, se requiere de una angiografía o una angio-resonancia magnética (RM) junto con un amplio diagnóstico diferencial de las posibles causas que puedan condicionar un MMS [5].

En el caso de la epilepsia en la MMA, no se requieren técnicas diagnósticas diferentes a las empleadas en el resto de pacientes, si bien se tiene que tener cuidado, especialmente en edad infantil, con la hiperventilación, al poder exacerbar los síntomas isquémicos. Se ha descrito que hasta el 50% de los pacientes pediátricos al hiperventilar muestran un patrón electroencefalográfico típico de esta entidad, conocido como patrón de *re-build up*. Dicho patrón consiste en la reaparición progresiva y anormal de ondas lentas (ritmo delta) tras un periodo inicial de normalización parcial del trazado electroencefalográfico por la hiperventilación. Esto es signo de la incapacidad de los vasos colaterales para mantener una perfusión normal tras el estrés metabólico que supone la hiperventilación ya que la hipocapnia se traduce a vasoconstricción cerebral [6].

Las guías actuales parecen enfocadas a controlar los síntomas isquémicos y hemorrágicos cerebrales, mientras que aspectos como la cefalea o la epilepsia han sido menos investigados. La epilepsia en la MMA puede suceder previo al ictus (típicamente el foco epileptógeno se encontrará en el territorio arterial estenosado), tras el ictus como epilepsia estructural o bien tras la cirugía debido a las nuevas dinámicas cerebrovasculares [7].

No se conoce cuál es el medicamento anticrisis (MAC) más apropiado para la MMA, y muchas veces se elige el fármaco por homología a otras epilepsias estructurales originadas por ictus [7].

El objetivo del presente estudio es aportar datos en vida real sobre la epilepsia en la MMA, especialmente por la falta de bibliografía en países occidentales, siendo el primer estudio europeo que abordaría esta problemática.

## 2. Material y Método

Se trata de un estudio observacional, retrospectivo y de base hospitalaria de los pacientes con MMA tratados en la red hospitalaria pública aragonesa. Se recuperaron todos los pacientes con diagnóstico de MMA entre enero de 1981 y diciembre de 2024 mediante los códigos CIE-9 437.5 y CIE-10 I67.5, sin excluirse pacientes con MMS.

Se consideró el diagnóstico de epilepsia siguiendo las recomendaciones de la Liga Internacional contra la Epilepsia (ILAE) en su documento de definición de la epilepsia de 2014 [8]. Quedan, por tanto, excluidas las crisis sintomáticas agudas (CSA) en el contexto de los primeros siete días tras un ictus al considerarse como un efecto transitorio del mismo [9].

Se recogieron múltiples variables sociodemográficas, comorbilidades, etiología en el caso de MMS, presencia de crisis epilépticas y el diagnóstico de epilepsia. Asimismo, se recogieron variables relacionadas con el tratamiento de estos pacientes, tanto de forma histórica como en el momento de redactar este artículo.

Para la recogida de datos se empleó *Microsoft Excel* de Office 2019 (Microsoft, Redmond, WA, USA) y para su posterior análisis se empleó la versión 2.4.11 de Jamovi (Jamovi Project, Sydney, Australia)

Se realizó un estudio descriptivo sobre los pacientes con y sin crisis de epilepsia y posteriormente se realizó un estudio comparativo analítico con el fin de poder establecer diferencias entre ambos grupos. Los pacientes únicamente con CSA en el contexto de un ictus (dentro de los primeros siete días evento cerebrovascular) no fueron considerados epilépticos para dicha comparación.

Debido a la baja población de pacientes con epilepsia (n = 11), se empleó el test de Fisher para realizar comparaciones entre variables dicotómicas y test no paramétricos para comparar variables numerales. Al tratarse de un estudio exploratorio, sobre un tema poco detallado en la bibliografía, se decidió no realizar una corrección de Bonferroni o de Benjamini-Hochberg inicialmente en aras a permitir establecer hipótesis para estudios futuros, si bien estos se añaden a los resultados obtenidos para su interpretación.

## 3. Resultados

Se recuperaron 26 pacientes, de los cuales destacan un fallecimiento y dos pacientes que residían fuera de la comunidad. Tomando la población de Aragón de 2024 según el censo del Instituto Aragonés de Estadística, se calculó una prevalencia de 1,71 casos por 100.000 habitantes, algo superior a estudios previos publicados en esta región [10, 11].

Con respecto al total de pacientes con MMA, la edad media al diagnóstico fue de 36,64 ± 18,07 años, y el 50% eran mujeres. El origen étnico más frecuente de la población fue el europeo con 16 pacientes (61,54%) seguido del este asiático con tres pacientes (11,54%, todos procedentes de China). Se muestran las características basales de la población en Tabla 1.

**Tabla 1.** **Tabla de la población a estudio, diferenciando entre pacientes con moyamoya con y sin epilepsia y comparativa entre ambos subgrupos**.

	Total de pacientes	Pacientes con MMA y epilepsia (n = 11)	Pacientes con MMA y sin epilepsia (n = 15)	*p* valor
Edad media al d(x) de la arteriopatía ± DE	36,64 ± 18,07	27,90 ± 16,80	43,10 ± 16,70	0,020
Edad media actual ± DE	44,76 ± 16,66	38,50 ± 14,20	49,30 ± 17,20	0,070
Sexo mujer (%)	13 (50,00%)	5 (45,45%)	8 (53,33%)	1,000
Fallecimiento	1 (3,84%)	0 (0,00%)	1 (6,67%)	1,000
HTA (%)	11 (42,31%)	4 (36,36%)	7 (46,67%)	0,701
DM (%)	4 (15,38%)	2 (18,18%)	2 (13,33%)	1,000
Tabaquismo (%)	9 (34,61%)	5 (45,45%)	4 (26,67%)	0,418
Dislipemia (%)	9 (34,61%)	3 (27,27%)	6 (40,00%)	0,682
ERC (%)	1 (3,84%)	1 (9,09%)	0 (0,00%)	0,423
Enfermedad arterial periférica (%)	1 (3,84%)	1 (9,09%)	0 (0,00%)	0,423
MMS (%)	12 (57,69%)	7 (63,63%)	5 (33,33%)	0,232
Afectación circulación anterior (%)	21 (80,76%)	7 (63,63%)	14 (93,33%)	0,128
Afectación circulación posterior (%)	7 (26,92%)	4 (36,36%)	3 (20,00%)	0,406
Origen europeo	15 (57,69%)	7 (63,63%)	8 (53,33%)	0,701
Intervención quirúrgica	11 (42,31%)	5 (45,45%)	6 (40,00%)	1,000
Ictus isquémico como inicio (%)	17 (65,38%)	9 (81,81%)	8 (60,00%)	0,216
Ictus hemorrágico como inicio (%)	3 (11,54%)	0 (0,00%)	3 (20,00%)	0,238
Ictus isquémico (%)	18 (69,23%)	10 (90,90%)	8 (60,00%)	0,083
Ictus hemorrágico (%)	5 (19,23%)	1 (9,09%)	4 (26,67%)	0,356
Síncope	5 (19,21%)	5 (45,45%)	0 (0,00%)	0,007
Cambio de personalidad	9 (34,61%)	6 (54,54%)	3 (20,00%)	0,103
Deterioro cognitivo	11 (42,31%)	8 (72,72%)	3 (20,00%)	0,014
Trastorno afectivo	10 (38,46%)	7 (63,63%)	3 (20,00%)	0,042
Cefalea	14 (53,84%)	7 (63,63%)	7 (46,67%)	0,452
Trastorno del movimiento	1 (3,81%)	0,00 (0,00%)	1 (6,67%)	1,000
Crisis epilépticas	13 (50,00%)	11 (100,00%)	2 (13,33%)	<0,001

DE, desviación estándar; DM, diabetes mellitus; ERC, enfermedad renal crónica; HTA, hipertensión arterial; MMA, angiopatía de moyamoya; MMS, síndrome de moyamoya.

Del total de pacientes, 12 (46,15%) presentaban un diagnóstico de MMS al identificarse una causa clara que produjera la entidad, siendo las más frecuentes la neurofibromatosis tipo 1 y las enfermedades autoinmunes (tres casos cada una).

Durante el transcurso de su enfermedad 13 (50%) pacientes habían padecido una crisis epiléptica, de los cuales 11 (42,31% del total) cumplían un diagnóstico formal de epilepsia según la ILAE. Solamente dos pacientes (7,69%) debutaron con crisis epilépticas como síntoma inicial.

Se adjunta en la Tabla 2 las particularidades sobre los pacientes con crisis a lo largo de su seguimiento, diferenciándose aquellos con CSA de los diagnosticados con epilepsia.

**Tabla 2.** **Pacientes con epilepsia y crisis sintomáticas agudas ILAE**.

N^o^	♀	EDAD D(X) MMA	EDAD ACT.	EDAD 1^a^ CRISIS	N^o^ FRCV	MMD o MMS	CAUSA MMS	ACV ISQ.	AIT	ACV HEM	EFR	N^o^ MAC	TIPO CRISIS	ETIOLOGÍA ILAE	CAUSA ETIOLOGÍA	EPILEPSIA ILAE	LÓBULO (SEMIOLOGÍA)	EE
	♂																	
1	♀	30,94	47,88	30,70	1	MMS	AI	1	0	0	0	2	F	Estructural	Ictus	Sí	Frontal	No
2	♂	7,22	19,38	0,72	0	MMS	Tr21	1	0	0	1	3	G	Genética	West Tr21	Sí	No localizador	No
3	♀	65,98	Fall.	n/d	1	MMD	-	1	0	1	0	0	D	CSA	Ictus	No	No localizador	No
4	♂	33,63	33,63	33,76	4	MMD	-	1	0	0	1	2	F	Estructural	Ictus	Sí	Frontal	No
5	♂	2,95	2,95	4,81	0	MMS	NF1	1	1	0	0	0	F	Genética	NF1	Sí	Frontal	No
6	♂	23,36	23,36	21,74	3	MMD	-	1	0	0	0	2	F	Estructural	Ictus	Sí	Temporal	No
7	♀	39,80	39,80	n/d	2	MMD	-	1	1	0	0	0	F	CSA	Ictus	No	Frontal	No
8	♂	54,75	Fall.	57,44	3	MMS	AI	1	0	1	1	2	F	Estructural	Ictus	Sí	Frontal	Sí
9	♀	7,17	7,17	5,17	0	MMD	-	0	1	0	0	0	F	Desconocida	¿?	Sí	Parietal	No
10	♀	49,84	49,843	43,36	3	MMD	-	0	1	0	1	4	F	Desconocida	¿?	Sí	Fronto-parietal	No
11	♀	32,82	32,82	32,77	1	MMS	NF1	1	0	0	0	2	F	Estructural, Genética	Ictus, NF1	Sí	No localizador	No
12	♂	30,18	30,18	30,10	0	MMS	RT	1	0	0	0	1	F	Estructural	Ictus, RT	Sí	Frontal	Sí
13	♀	33,56	33,56	27,25	0	MMD	-	1	0	0	0	1	F, G	Estructural	Ictus	Sí	No localizador	No

ACV, accidente cerebrovascular; ISQ, Isquémico; HEM, Hemorrágico; AI, autoimmune; AIT, accidente isquémico transitorio; CSA, crisis sintomática aguda; D(X), diagnóstico; EE, estatus epiléptico; EFR, epilepsia fármaco resistente (según ILAE); FRCV, factores de riesgo cardiovascular (al ingreso); ILAE, International League Against Epilepsy; MMD, enfermedad de moyamoya (moyamoya disease); n/d, no disponible; NF-1, neurofibromatosis tipo I; RT, radioterapia; Tipos de crisis: F/G/D, focal, generalizada, de inicio desconocido.

Los pacientes con diagnóstico de epilepsia presentaban una edad al diagnóstico de 27,9 ± 16,8 años, cinco (45,45%) eran mujeres y el origen étnico más frecuente fue el europeo (siete pacientes, el 63,63%). De los once pacientes con diagnóstico de epilepsia, seis (54,54%) había sido diagnosticado de MMS. El tiempo de seguimiento mediano de su epilepsia fue de 8,11 años (RIC 4,86–15,30). Cinco de los 11 pacientes (45,45%) fueron intervenidos quirúrgicamente, tres con técnica indirecta, uno con técnica directa y otro con técnica mixta. Ninguno de los pacientes debutó con epilepsia tras ser intervenido.

Con respecto a la clasificación operativa de la epilepsia [12], nueve de los 11 pacientes presentaban una epilepsia focal, y de los dos sobrantes, uno fue diagnosticado de epilepsia de origen indeterminado y el otro, de epilepsia generalizada. Atendiendo a su etiología, ocho presentaron una causa estructural, un paciente un síndrome epiléptico (síndrome de West asociado a síndrome de Down) y en dos pacientes no se estableció una causa clara, al presentar una resonancia sin alteraciones estructurales.

Todos los pacientes fueron estudiados con electroencefalografía. Ningún electroencefalográfico fue realizado de forma ictal.

Los dos pacientes que presentaron un estatus epiléptico presentaron un patrón electroencefalográfico compatible ictal. El trazado electroencefalográfico registrado fue de estatus epiléptico generalizado en ambos casos. Con respecto a la clasificación clínica del estatus, este fue un estatus epiléptico convulsivo de inicio focal con evolución a bilateral. En uno de los casos el estatus fue la causa del fallecimiento del paciente.

Dos pacientes presentaron focos interictales compatibles, uno de ellos durante su etapa infantil (síndrome de West), que posteriormente evolucionó a una posible epilepsia estructural. Sin embargo, hasta en siete casos, aquellas crisis consideradas como focales presentaban focos de lentificación post-ictal coherentes con la semiología aportada. En los casos en los que se evidenció lentificación interictal, hubo una correlación con la lesión topográfica solamente en los casos en los cuales el origen de la epilepsia estructural se debía a un ictus isquémico.

Atendiendo a la semiología por lóbulos, la más frecuente fue la asociada al lóbulo frontal, ya que hasta ocho de los pacientes presentaron crisis de inicio motor (no automatismos), sin poder manifestar la existencia de síntomas no observables previos al fenómeno motor. Cinco pacientes presentaban crisis de semiología parietal (de los cuales cuatro presentaban crisis motoras como las previamente descritas).

**Fig. 1.**
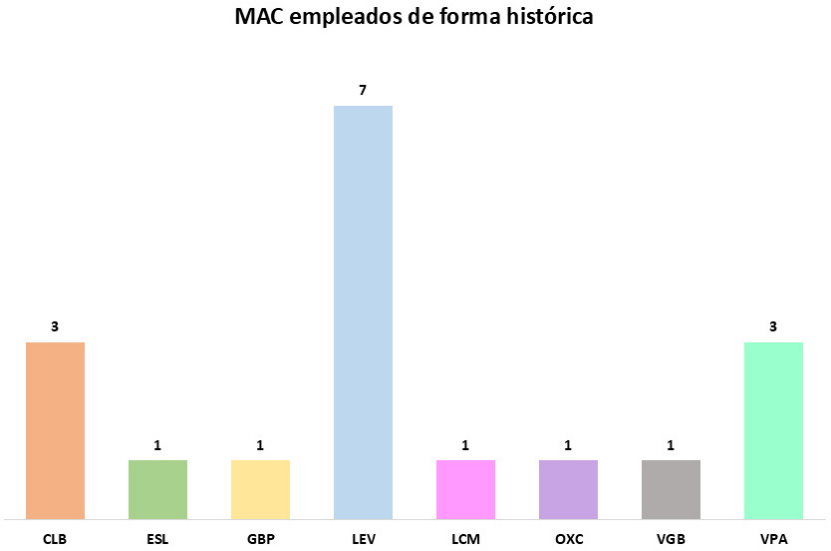
**Medicamentos anticrisis histórico**. MAC, medicación anticrisis; CLB, clobazam; ESL, eslicarbazepina; GBP, gabapentina; LEV, levetiracetam; LCM, lacosamida; OXC, oxcarbacepina; VGB, vigabatrina; VPA, valproato.

**Fig. 2.**
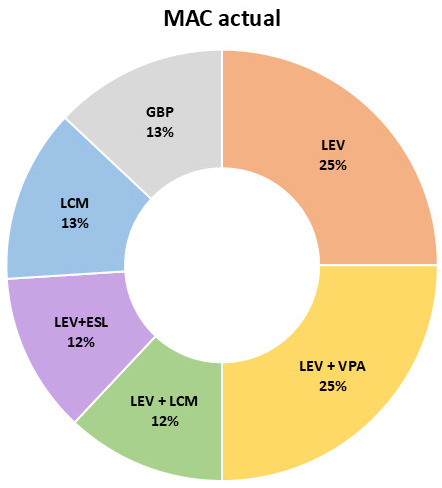
**Medicamentos anticrisis actual**. ESL, eslicarbazepina; GBP, gabapentina; LCM, lacosamida; VGB, vigabatrina.

Con respecto al tratamiento más frecuentemente usado en el momento de la redacción de este artículo, el levetiracetam fue empleado en monoterapia o biterapia en seis de los once pacientes; solo dos de ellos no recibieron tratamiento (uno de ellos por debut en edad infantil y posterior control y otro de ellos por efectos adversos). Centrándose en el tratamiento histórico con MACs, los fármacos más empleados a lo largo del seguimiento de los pacientes fueron levetiracetam (7/11 pacientes) seguido del ácido valproico y del clobazam (3/11 pacientes en ambos casos). Se adjunta Fig. 1 donde se muestran los MACs usados a lo largo del seguimiento y el Fig. 2 que hace referencia a los MACs prescritos en el momento de la redacción.

Del total de los pacientes con diagnóstico de epilepsia 4/11 (36,36%) presentaban criterios de epilepsia fármaco-resistente. Hubo dos casos de MMS y dos de MMD. Los estudios realizados no difirieron del resto de pacientes (angiografía diagnóstica y RM con angioRM en el seguimiento). En tres de los casos, como se muestra en la Tabla 2, hubo un ictus isquémico durante su seguimiento. En dos de los casos la topografía de la crisis coincidía con la del ictus isquémico. Sin embargo, la topografía de las lesiones isquémicas no explicó en la totalidad de casos el origen supuesto de la crisis por semiología. En la paciente 10 aunque las crisis fueran focales, no había en la RM signos de un infarto isquémico previo y para garantizar el diagnóstico se realizaron varios EEG que mostraron grafoelementos epileptiformes en regiones frontales con predominio derecho.

En ninguno de los pacientes, ni siquiera en los pediátricos, se encontró el “*re-build up phenomenon*” electroencefalográfico, a pesar de que en casi todos se realizaron maniobras de hiperventilación durante el estudio.

Realizando una comparativa entre pacientes con y sin epilepsia (recogidas en Tabla 1) se evidenciaron diferencias estadísticamente significativas (*p*-valor < 0,05) en la edad de diagnóstico de la MMA, la presencia de deterioro cognitivo, síncopes, trastorno afectivo y crisis epilépticas. La presencia de deterioro cognitivo, síncopes y trastorno afectivo fueron más frecuentes entre los pacientes con epilepsia y MMA. Se encontró tendencia a la significación estadística (*p*-valor < 0,10) en la presencia de ictus isquémico, con mayor porcentaje en los pacientes con epilepsia. No se encontraron diferencias en la presencia de ictus hemorrágico, el tipo de circulación afectada, el porcentaje de MMS, origen étnico o el inicio en forma de ictus isquémico o hemorrágico.

Si se aplica las correcciones de Bonferroni, al realizarse 26 comparaciones, ninguna de las variables alcanza el *p*-valor para el ajuste del error de tipo I (0,0019) salvo en la presencia de crisis epilépticas.

## 4. Discusión

Existe escasa bibliografía que aborde la epilepsia en los pacientes con MMA y cómo esta afecta su calidad de vida o pronóstico. Además, la mayor parte de la bibliografía trata sobre pacientes pediátricos, de origen asiático o casos de pacientes intervenidos quirúrgicamente [13, 14].

Atendiendo a los resultados epidemiológicos de nuestra serie con respecto a series asiáticas, destaca la elevada prevalencia de MMS, siendo del 50% en nuestra serie y en torno al 10% en las series referenciadas. El aumento del porcentaje de MMS con respecto al total también se ha evidenciado en otras series realizadas en regiones occidentales, si bien en estos estudios la forma de diferenciar entre MMS o MMD ha sido de forma heterogénea y aplicando criterios cambiantes [3, 15, 16].

El porcentaje de pacientes con epilepsia (42,31%) o incluso con crisis epilépticas (50%) dista mucho de lo encontrado en otras series, como la serie de Xiang-Yang donde de 470 pacientes quirúrgicos de procedencia china, solo 4 (0,9%) habían presentado crisis epilépticas -sin diferenciar si se diagnosticaron o no de epilepsia- [17]. En comparativa con dicha serie, también se evidenció mayor prevalencia de cefalea en nuestra serie (53,8% vs 9,6%) y de ictus isquémico (73,1% vs 26,8%) lo que podría apoyar la teoría de la existencia de un fenotipo occidental de la angiopatía, con diversas manifestaciones clínicas y mayor porcentaje de MMS [3]. Sin embargo, en nuestra serie no se evidenciaron diferencias estadísticamente significativas en el origen étnico de los pacientes en función de si presentaban o no diagnóstico de epilepsia.

Comparando con la serie de Starke *et al*. [18] realizada en Estados Unidos, hasta el 14% de sus pacientes habían presentado crisis epilépticas previas a la cirugía -sin determinar si se trataban de CSA o permitían el diagnóstico formal de epilepsia-. En este caso la prevalencia de ictus isquémicos fue del 61%, algo más próxima a nuestra serie.

No obstante, parece haber cierta relación con las crisis epilépticas y las cirugías de revascularización cerebral. En caso de pacientes pediátricos, la prevalencia de crisis antes de un tratamiento quirúrgico podría ser de hasta un 18,1% [19]. En el caso de los adultos, hasta el 18,9% de estos podrían presentar crisis tras la cirugía, y un 6% podrían presentar epilepsia tras la cirugía [20].

Otras fuentes bibliográficas señalan que la epilepsia tiene una prevalencia en vida del 5%–20% en los pacientes con MMA [2, 14], lo que implica una importante variabilidad en su prevalencia y que es bastante inferior a lo reportada en este estudio. Tal vez ello pueda estar relacionada con un aumento del porcentaje del ictus en nuestra serie, o incluso al mismo fenotipo occidental donde las causas secundarias de MMA son más frecuentes.

En relación con los factores predictivos para la epilepsia en pacientes con MMA, el estudio de Mikami *et al*. [14] identificó diferencias estadísticamente significativas en la edad de inicio de la enfermedad, la presencia de ictus hemorrágico previo, la presencia de ictus isquémico de gran tamaño medido mediante el *CVA-score* y la presencia de crisis agudas sintomáticas en contexto del ictus inicial.

En nuestro estudio, aunque no se hace un análisis sobre factores predictivos, se encuentran diferencias estadísticamente significativas entre los pacientes con MMA que padecen epilepsia y los que no en la edad de inicio de la arteriopatía y la presencia de ictus isquémico, pero no en la presencia de ictus hemorrágicos. Sin embargo, existen algunos aspectos que dificultan la comparativa de nuestro estudio con el de Mikami. En primer lugar, el estudio de Mikami analiza la clínica al inicio del diagnóstico, mostrando un 12,5% de ictus isquémicos frente a nuestro 73,1% durante el seguimiento de la enfermedad. En segundo lugar, la elevada prevalencia de ictus isquémicos en nuestra serie hace pensar que al ser una entidad poco conocida en nuestro entorno solo se han diagnosticado aquellos casos con una manifestación clínica más grave.

Existe poca bibliografía al respecto de qué fármacos emplear, recomendándose fármacos para crisis focales, si bien aquellos de amplio espectro como el levetiracetam o el valproato han sido empleados con éxito [7]. No se ha encontrado en la bibliografía ningún artículo que aborde qué fármaco se emplea más a menudo o si existen efectos adversos más frecuentes en estos pacientes ante la exposición a fármacos anticrisis.

Se estima que el porcentaje de pacientes epilépticos con epilepsia fármaco resistente (EFR) podría encontrarse en torno al 30% siendo más frecuente cuando existe una causa estructural [21]. Se ha descrito que el porcentaje de EFR debida al ictus -isquémico o hemorrágico- es del 18,2%–19,5% [22, 23]. Por lo que podrían ser otras causas estructurales las que elevan el riesgo de fármaco-resistencia, si bien esto debería ser explorado en estudios futuros [21]. En nuestro caso, de 18 pacientes con MMA con ictus durante su seguimiento, tres presentaron criterios de EFR (3/18, 16,66%). Solo uno de los pacientes con diagnóstico de EFR no había presentado un ictus durante su seguimiento.

Analizando una de las pocas series que evalúa el tipo de crisis, coincide que en ambos casos el tipo de crisis más frecuente fue la focal (76,92% del total de las crisis en nuestra serie frente al 73,91% de la serie de Nakase) [24].

Se adjunta en la Tabla 3 [14, 19, 25, 26, 27, 28, 29, 30, 31, 32, 33, 34, 35, 36] los principales trabajos indexados en los últimos diez años sobre epilepsia asociada a MMA encontrados en la bibliografía, atendiendo a su manejo y si existe diagnóstico de EFR. En dicha tabla se hace hincapié en el escaso conocimiento sobre la EFR en la MMA, el escaso conocimiento sobre los estatus epilépticos en esta enfermedad y la ausencia de variedad de estudios que aborden el tratamiento anticrisis empleado [14, 19, 25, 26, 27, 28, 29, 30, 31, 32, 33, 34, 35, 36].

**Tabla 3.** **Casos o series publicadas sobre pacientes con MMA y epilepsia**.

Autor y año. Referencia	Pacientes con epilepsia y MMA	Tipo de pacientes	País	N^o^ de pacientes con MMS	N^o^ de pacientes con epilepsia focal	N^o^ de pacientes con epilepsia estructural	N^o^ de pacientes con EFR	N^o^ de pacientes con cirugía de revascularización cerebral	Fármacos empleados fuera de EE o profilaxis quirúrgica (%)	N^o^ pacientes con EE
Viteva *et al*. (2024) [25]	1	Caso clínico	Bulgaria	0	1	1	0	0	VPA	0
Alotaibi *et al*. (2024) [26]	1	Caso clínico	Arabia Saudí	1	0	n/d	0	0	n/d	1
Mikami *et al*. (2015) [14]	7	Serie quirúrgica	Japón	0	4	7	1	7	VPA (42,85%)	n/d
									LEV (28,57%)	
									PHT (28,57%)	
									ZNS (14,29%)	
									CLB (14,29%)	
Liu *et al*. (2023) [27]	26	Serie quirúrgica	China	0	n/d	n/d	n/d	26	n/d	n/d
Gatti *et al*. (2023) [29]	34	Serie pediátrica	Estados Unidos	13	10	32	n/d	n/d	n/d	n/d
Abdul Rab *et al*. (2023) [28]	1	Caso clínico	Arabia Saudí	1	1	1	1	0	VPA, LEV, CLB	1
Das *et al*. (2023) [30]	1	Caso clínico	India	n/d	1	1	0	0	OXC	n/d
Alramadan *et al*. (2021) [31]	11	Serie quirúrgica	Arabia Saudí	10	n/d	n/d	n/d	11	n/d	n/d
Talbot *et al*. (2020) [32]	1	Caso clínico	Reino Unido	1	0	0	0	0	VPA, LEV	n/d
Lu *et al*. (2020) [33]	17	Serie de casos	China	0	11	n/d	n/d	n/d	n/d	n/d
Nakayama *et al*. (2019) [34]	1	Caso clínico	Japón	0	1	0	0	1	LEV	0
Garson *et al*. (2018) [36]	1	Caso Clínico	Estados Unidos	1	1	0	1	1	LEV, LTG, CLB	0
Kuroda *et al*. (2019) [35]	1	Caso clínico	Japón	1	1	1	1	1	n/d	0
Ma *et al*. (2018) [19]	28	Serie de casos infantiles prequirúrgica	China	0	19	9	n/d	28	n/d	n/d

Tabla 3: se muestran los casos y series de casos publicadas en la bibliografía que abordan la cirugía en pacientes con epilepsia; PHT, fenitoína; ZNS, zonisamida; n/d, no disponible.

En ninguno de nuestros pacientes se evidenció el fenómeno de *rebuild-up*. Sin embargo, en el artículo de Kodama que describe los patrones electroencefalográficos en pacientes pediátricos con MMA se evidenció que al menos el 58,33% de los pacientes presentaban este fenómeno [37]. No obstante, el estudio de Lu *et al*. [33] con 17 pacientes pediátricos con epilepsia y MMA solamente encontró un caso de “*re-build up*”, por lo que este fenómeno podría ser menos frecuente de lo inicialmente descrito. En el caso de la serie de Frechette *et al*. [38] de pacientes adultos con MMA no mostró ningún caso de fenómeno de *rebuild-up*. En dicho estudio se refleja que la alteración electroencefalográfica más frecuente en estos pacientes es la lentificación focal en un 78,6%.

Como principales limitaciones a nuestro estudio, destaca la escasa población, al tratarse de un estudio en una única región y de una enfermedad con una baja prevalencia en países occidentales. Asimismo, el hecho de explorar una complicación poco estudiada y no universal a todos los pacientes con MMA implica que los grupos de comparación sean todavía más pequeños. Asimismo, se trata de un estudio retrospectivo, por lo que puede existir sesgos a la hora de la recogida de los datos. Ello implica que este estudio solo pueda pretender establecer hipótesis para estudios futuros, sin embargo, dada la escasez de bibliografía sobre el tema, especialmente en entornos occidentales, hace relevante la publicación de los datos.

## 5. Conclusiones

La epilepsia en la MMA en nuestra población es mayor a la reportada en otras series, si bien las diferencias con respecto a edad y entorno en el que se han realizado estos estudios podrían dificultar su comparativa.

La presencia de epilepsia en la MMA podría ser superior en los pacientes que padecen síncopes, trastorno afectivo, deterioro cognitivo y una edad menor al diagnóstico de la enfermedad.

No hay evidencia suficiente sobre la eficacia y seguridad de los fármacos anticrisis en pacientes con MMA. Tampoco se dispone de datos sobre la prevalencia de epilepsia fármaco-resistente en esta población ni sobre qué tipo de cirugía (revascularización o específica para epilepsia) resulta más beneficiosa.

Se requiere más estudios que aborden esta comorbilidad de la enfermedad, que investiguen sus consecuencias y que aporten datos en vida real sobre cuál es la mejor estrategia terapéutica para el control de la epilepsia.

## Data Availability

Los conjuntos de datos utilizados y analizados durante el presente estudio están disponibles a través del autor de correspondencia (Mario Bautista-Lacambra, mariobautistalacambra@gmail.com) previa solicitud razonable. Siempre se salvaguardará la privacidad e intimidad relativa a los pacientes de los cuales estos emanan.

## Divulgación

A small sample of the descriptive work was shown on the LXXVI Reunión Anual de la Sociedad Española de Neurología, analyzing epidemiological aspects of epilepsy in moyamoya angiopathy and how it had been managed pharmacologically in our centers.

## Contribuciones de los Autores

MBL, VGI, LFTR, MSM, JMH, ALL, RBC, CTJ, MPL, HTM han formado parte en el proceso de diseño, corrección y aceptación del siguiente manuscrito. MBL, VGI y HTM han participado en la redacción del mismo. Todos los autores leyeron y aprobaron el manuscrito final. Todos los autores han participado lo suficiente en el trabajo y han acordado ser responsables de todos los aspectos del mismo.

## Aprobación Ética y Consentimiento Informado

El protocolo de investigación fue aprobado por el Comité de Ética de Aragon Clinical Research Ethics Committee (CEICA): (Número de Aprobación Ética: C.P. - C.I. PI22/309), y todos los participantes proporcionaron consentimiento informado firmado. El estudio se llevó a cabo de acuerdo con las directrices de la Declaración de Helsinki.

## Agradecimientos

En agradecimiento al Grupo de Estudio de Enfermedades Cerebrovasculares de la Sociedad Aragonesa de Neurología.

## Financiación

Esta investigación no recibió financiación externa.
